# Amlexanox and Forskolin Prevents Isoproterenol-Induced Cardiomyopathy by Subduing Cardiomyocyte Hypertrophy and Maladaptive Inflammatory Responses

**DOI:** 10.3389/fcell.2021.719351

**Published:** 2021-09-24

**Authors:** Gabriel Komla Adzika, Hongjian Hou, Adebayo Oluwafemi Adekunle, Ruqayya Rizvi, Seyram Yao Adzraku, Kexue Li, Qi-Ming Deng, Richard Mprah, Marie Louise Ndzie Noah, Joseph Adu-Amankwaah, Jeremiah Ong’achwa Machuki, Wenkang Shang, Tongtong Ma, Stephane Koda, Xianluo Ma, Hong Sun

**Affiliations:** ^1^Department of Physiology, Xuzhou Medical University, Xuzhou, China; ^2^The College of Biology and Food, Shangqiu Normal University, Shangqiu, China; ^3^Xuzhou Medical University, Xuzhou, China; ^4^Key Laboratory of Bone Marrow Stem Cell, Department of Hematology, The Affiliated Hospital of Xuzhou Medical University, Xuzhou, China; ^5^The Key Laboratory of Cardiovascular Remodeling and Function Research, The State and Shandong Province Joint Key Laboratory of Translational Cardiovascular Medicine, Chinese Ministry of Education, Department of Cardiology, Chinese National Health Commission and Chinese Academy of Medical Sciences, Qilu Hospital of Shandong University, Jinan, China; ^6^Faculty of Biology, Institute of Biochemistry and Molecular Biology, ZBMZ, Albert-Ludwigs University of Freiburg, Freiburg, Germany; ^7^Laboratory of Infection and Immunity, Department of Pathogenic Biology and Immunology, Xuzhou Medical University, Xuzhou, China; ^8^Internal Medicine—Cardiovascular Department, People’s Hospital of Jiawang District, Xuzhou, China

**Keywords:** chronic catecholamine stress, amlexanox, forskolin, GRK5, adenylyl cyclase, cAMP, inflammation, isoproterenol-induced cardiomyopathy

## Abstract

Chronic catecholamine stress (CCS) induces the occurrence of cardiomyopathy—pathological cardiac hypertrophy (PCH), which is characterized by left ventricular systolic dysfunction (LVSD). Recently, mounting evidence has implicated myocardial inflammation in the exacerbation of pathological cardiac remodeling. However, there are currently no well-defined treatment interventions or regimes targeted at both the attenuation of maladaptive myocardial hypertrophy and inflammation during CCS to prevent PCH. G protein-coupled receptor kinase 5 (GRK5) and adenylyl cyclases (ACs)-cAMP mediates both cardiac and inflammatory responses. Also, GRK5 and ACs are implicated in stress-induced LVSD. Herein, we aimed at preventing PCH during CCS via modulating adaptive cardiac and inflammatory responses by inhibiting GRK5 and/or stimulating ACs. Isoproterenol-induced cardiomyopathy (ICM) was modeled using 0.5 mg/100 g/day isoproterenol injections for 40 days. Alterations in cardiac and inflammatory responses were assessed from the myocardia. Similarities in the immunogenicity of cardiac troponin I (cTnI) and lipopolysaccharide under CCS were assessed, and Amlexanox (35 μM/ml) and/or Forskolin (10 μM/ml) were then employed *in vitro* to modulate adaptive inflammatory responses by inhibiting GRK5 or activating ACs-cAMP, respectively. Subsequently, Amlexanox (2.5 mg/100 g/day) and/or Forskolin (0.5 mg/100 g/day) were then translated into *in vivo* during CCS to modulate adaptive cardiac and inflammatory responses. The effects of Amlexanox and Forskolin on regulating myocardial systolic functions and inflammatory responses during CCS were ascertained afterward. PCH mice had excessive myocardial hypertrophy, fibrosis, and aggravated LVSD, which were accompanied by massive CD68^+^ inflammatory cell infiltrations. *In vitro*, Forskolin-AC/cAMP was effective than Amlexanox-GRK5 at downregulating proinflammatory responses during stress; nonetheless, Amlexanox and Forskolin combination demonstrated the most efficacy in modulating adaptive inflammatory responses. Individually, the translated Amlexanox and Forskolin treatment interventions were ineffective at subduing the pathological remodeling and sustaining cardiac function during CCS. However, their combination was potent at preventing LVSD during CCS by attenuating maladaptive myocardial hypertrophy, fibrosis, and inflammatory responses. The treatment intervention attained its potency mainly *via* Forskolin-ACs/cAMP-mediated modulation of cardiac and inflammatory responses, coupled with Amlexanox inhibition of GRK5 mediated maladaptive cascades. Taken together, our findings highlight the Amlexanox and Forskolin combination as a potential therapeutic intervention for preventing the occurrence of pathological cardiac hypertrophy during chronic stress.

## Background

The elevated level of circulating catecholamines during chronic stress is a hallmark for the initiation of adverse remodeling of the left ventricular (LV) (Frey et al., 2004; Liu et al., 2015; Zhang et al., 2019). The progression in LV remodeling has been demonstrated to result in irreversible pathological cardiac hypertrophy (PCH) if there are no timely preventive measures to subdue the excessive firing of neurohormonal stimuli during chronic stress (Schaeuble et al., 2019; Zhang et al., 2019). In recent studies, irreversible hypertrophic cardiomyopathy has been associated with LV systolic dysfunction (LVSD) (Marstrand et al., 2020). Also, the resulting LVSD has been attributed to the distortion of the typical myocardial architecture due to increased LV wall thickness and fibrosis (Galati et al., 2016; Marstrand et al., 2020), thereby making the heart incapable of rapidly replenishing blood for the subsequent ejections (Xie et al., 2013). Although the mechanisms underlying the cardiomyopathy are still being elucidated, the most recent findings have implicated hyperactive myocardial proinflammation responses as a factor hastening the pathological remodeling (Zhao et al., 2017; Weisheit et al., 2021).

In steady state, cardiac resident macrophages have been shown to play crucial roles in reparative functions after cardiac injuries (Heidt et al., 2014). However, an enormous amount of cardiomyocyte deaths (*via* both apoptosis and necrosis) occur during chronic catecholamine stress (CCS) (Whelan et al., 2010; Liu et al., 2015). The apoptotic myocyte directly induces fibrosis (Liu et al., 2015), while troponins released from the necrotic cardiomyocytes serve as a damage-associated molecular pattern that invokes proinflammatory responses from macrophages in an attempt to curb further deaths (Haider and Stimson, 1993; Kaya et al., 2008; Heidt et al., 2014). However, under CCS conditions, prolonged secretion of proinflammatory cytokines; interleukin (IL) 1β, IL-2, IL-6, tumor necrosis factor-alpha (TNFα), and interferon-gamma (IFN-γ) results, while the anti-inflammatory cytokines; IL-10, and transforming growth factor-beta (TGF-β) secretions are downregulated (Kaya et al., 2010). The upregulated secretions of IL-6 and TNFα prolong myofibroblast activations and increases interstitial fibrosis markedly, which stiffens the myocardia and causes LVSD (Kaya et al., 2010; Hartupee and Mann, 2016; Hulsmans et al., 2016).

Elucidation of the pathomechanisms underlying the occurrence of PCH has shown the involvements of β-adrenergic receptors (βARs) and G protein-coupled receptor kinases 5 (GRK5) activities in both cardiomyocytes and immune cells (Hullmann et al., 2014; Adzika et al., 2019). Stimulation of βARs by physiological levels of catecholamines inducing signaling *via* the stimulatory G protein (G_αs_)—adenylyl cyclases (ACs)—cyclic adenosine monophosphate (cAMP) pathway (Adzika et al., 2019). In cardiomyocytes, AC5/AC6-cAMP—L-type calcium channel (LTCC) facilitates inotropic and chronotropic, while in immune cells, AC7-cAMP exerts adaptive immunoregulation on transcriptional factors (TFs) as such; nuclear factor of activated T-cells (NFATs), and NF-kB (Paur et al., 2012; Raker et al., 2016; Murphy et al., 2019). As such, ACs-cAMP activities are essential for sustaining the myocardial function under physiological states. However, during CCS, signaling *via* the G_αs_/ACs/cAMP pathway is impeded, affecting its modulation of cardiac and immune functions.

GRKs modulate the expression and activities of βARs. While GRK2 engages in βARs desensitizes, GRK5 phosphorylates the receptors to initiate G protein-coupled receptors (GPCR)-independent signaling, which induces PCH (Gold et al., 2012; Hullmann et al., 2014; Traynham et al., 2016). Also, GRK5 translocations into nuclei to maladaptively phosphorylate cardiac hypertrophy and inflammatory TFs; NFATs, myocyte enhancer factor 2 (MEF2), GATA4, and NF-kB hastens the pathological remodeling (Hullmann et al., 2014).

Currently, there are no stipulated treatment regimens that aim at attenuating PCH during CCS through the simultaneous prevention of maladaptive myocardial hypertrophy, fibrosis, and inflammation. Herein, we demonstrate that during chronic isoproterenol-induced cardiomyopathy (referred to as PCH hereafter), the expression of βARs, ACs, GRKs, cardiac hypertrophy, and inflammatory TFs are altered. Also, we showed that inflammatory responses elicited by troponin in the PCH hearts were similar to those induced when its activity was mimicked *in vitro* with lipopolysaccharides (LPS) during stress.

GRK5 and ACs were targeted with amlexanox (ALX) and forskolin (FSK), respectively, to explore PCH treatment interventions. ALX has been shown to inhibit GRK5-mediated hypertrophy in PCH models; however, there is no evidence that cardiac functions were preserved (Homan et al., 2014). Also, ALX is demonstrated to inhibit inflammatory responses activated *via* GRK5 (Patial et al., 2011; Quan et al., 2019). The stimulation of ACs by FSK facilitates the bioavailability of cAMP (Huang and Wong, 1989; Chiadak et al., 2016), which might ensure the modulation of cardiac and inflammatory functions during CCS. Therefore, by inhibiting GRK5 and stimulating ACs, we attenuated the hyperactive proinflammatory responses observed *in vitro*. Furthermore, these treatment interventions were translated *in vivo* to assess their therapeutic potential in attenuating PCH through modulation inflammatory and hypertrophy responses.

## Materials and Methods

### Experimental Animals and Models

Male FVB mice aged between 8 and 12 weeks were randomized into groups (*n* = 4–16 mice per group depending on the experimental setting) and used for both *in vitro* and *in vivo* experiments. Isoproterenol-induced cardiomyopathy models were made by subcutaneous injections of isoproterenol (160504; Sigma) 0.5 mg/100 g/day as previously demonstrated (Lin et al., 2016; Zhou et al., 2017), for 40 days. The equivalents of the placebo (5% v/v dimethyl sulfoxide) were injected to the vehicle (Vhl) group. Also, because it has been shown that laboratory animal handling induces background stress (Gouveia and Hurst, 2019), littermates without any handling were included in the study and designated as the control (Ctrl) group to rule out handling as a contributor to any observed pathological alterations.

PCH preventive models were made by intraperitoneal injection of ALX (ab142825; Abcam) 2.5 mg/100 g/day and/or FSK (1099; Tocris Bioscience, United Kingdom) 0.5 mg/100 g/day. These treatments were administered during the isoproterenol-induced cardiomyopathy modeling (Supplementary Figure 1).

The mice were euthanatized by cervical dislocation, and macrophages or the hearts were isolated for further analysis.

### Electrocardiography and Echocardiography

Mice, 10–11 per treatment group, were mildly sedated with 0.5% isoflurane. The mice were fixed with echo gel, and their body temperatures were monitored and maintained at 37°C for electrocardiography (ECG) data acquisition with Vevo 2100 Ultrasound system (VisualSonics, Toronto, Canada). Simultaneously, global cardiac function was assessed in M-mode with a Vevo 2100 Ultrasound system (VisualSonics, Toronto, Canada). At end-systole and end-diastole, the interventricular septal wall and posterior wall thicknesses, as well as left ventricle internal diameters, were evaluated from the left parasternal longitudinal axis view. Ejection fraction (EF) and fractional shorten (FS) were calculated as described (Jia et al., 2019).

### Immune Cell Isolation and Culture

Peritoneal macrophages (PM_ϕ_) (*n* ≥ 2 ^∗^ 10^6^ cells per treatment group) were isolated from mice as previously described (Ray and Dittel, 2010) but with some modifications. Eight to ten milliliters of 37°C PBS containing 3% fetal bovine serum (FBS) was carefully injected into the peritoneal cavity. The cell suspensions were then collected from the peritoneum after 5 min and centrifuged at 1,500 rpm for 10 min. The pellets of cells were resuspended in 10% FBS and cultured for 48 h. The cells were initially identified using F4/80 (123116; BioLegend) and CD11b (101206; BioLegend). Subsequently, cultured PM_ϕ_ were pre-treated for 1 h with 35 μM/ml of ALX, and challenged with 10 μM/ml of ISO and/or 1 μg/ml LPS along with the same dose of ALX treatment for 24 h. Also, PM_ϕ_ challenged with ISO and LPS was treated with 10 μM/ml FSK. The combination of ALX and FSK treatment was employed as well. The supernatants were used for ELISA assays, while the PM_ϕ_ were used to assess GRK5 activities *in vitro*.

### Enzyme-Linked Immunosorbent Assay

Sera and lysate from *in vivo* models (*n* = 7–9 mice per treatment group) and cell culture media supernatants from treated PM_ϕ_ were used to assess the concentrations of proinflammatory cytokines (IL-1β, IL-6, and TNFα), anti-inflammatory cytokines (IL-10 and TGF-β), troponin I and cAMP. IL-1β (ab197742; Abcam), IL-6 (ab222503; Abcam), TNFα (ab208348; Abcam), IL-10 (ab255729; Abcam), TGF-β (KE10005; Proteintech), cAMP (JL13362; Jianglai Bio, China), and troponin I (JL31923; Jianglai Bio, China) ELISA were performed in triplicates as per the instructions of the manufacturer.

### RNA Extraction and Real-Time qPCR

Total RNA was extracted from ventricular apical myocardium (*n* = 6 hearts per treatment group) using TRIzol (15596026; Life technologies). cDNA was synthesized from 1 μg of RNA using a reverse transcription kit according to the instruction of the manufacturer (FSQ107; Toyobo). The obtained cDNAs were amplified by semiquantitative RT-PCR using SYBR Green Master Mix (Q111-02; Vazyme). The primer sequences used are listed here (Supplementary Table 1). The relative gene expressions were normalized to the expressions of GAPDH and were compared as described (Gold et al., 2012).

### Immunofluorescence Staining

Cultured and pre-treated PM_ϕ_ (*n* ≥ 2 ^∗^ 10^6^ cells per treatment group) were fixed and permeabilized with pre-chilled methanol–acetone (ratio 1:1). Non-specific antibody binds were blocked with 1% BSA in PBS for 1 h. PM_ϕ_ were then incubated with GRK5 primary antibody (ab64943; Abcam) at 4°C overnight, washed with PBS, and probed with R-PE-conjugated secondary antibody (SA00008-2; Proteintech) at room temperature for 1 h. Next, cells were washed with PBS, conditioned with 0.5% BSA in Hanks’ balanced salt solution, and the cytoplasmic membranes were stained with cholera toxin B (CTxB) (C34775, Thermo Fisher Scientific) for 30 min at 4°C. DAPI nuclei staining were done and followed by imaging and assessment of the ratios of nucleic and cytoplasmic GRK5 expressions with ImageJ (*n* = 12–15 cells per four mice).

### Histological Analysis of Hypertrophy, Interstitial Fibrosis, and Immune Cells Infiltration

Hearts were excised (*n* = 6–10 mice per treatment group), rinsed, and fixed in 10% formaldehyde overnight. The hearts were then embedded in paraffin and sectioned at 4 μm. Subsequently, H&E (G1120; Solarbio), Masson’s trichrome (G1340; Solarbio), wheat germ agglutinin (WGA) (W11261; Thermo Fisher Scientific), and CD68 (ab955; Abcam) immunohistochemical staining were then performed as previously described (Qi et al., 2019). Imaging of tissue slides was done at × 400 magnification and analyzed with ImageJ (1.52a version; National Institute of Health, United States).

### Western Blot

Apical myocardia harvested from four to six hearts per treatment group were homogenized in a lysis buffer containing phosphatase and proteinase cocktail inhibitor, in a ratio of 100:1:1, respectively. Lysates of normalized concentrations treated with reducing agents were denatured at 100°C for 10 min and separated on SDS-PAGE gels. The transferred protein bands were blocked and immunoblotted overnight in the following primary antibodies: β_1_AR (ab3442; Abcam), β_2_AR (ab182136; Abcam), MEF2 (ab64644; Abcam), GRK5 (ab64943; Abcam) GATA4 (ab84593; Abcam), NFAT (ab25916; Abcam), AC5 (PAC-501AP; FabGennix), AC6 (PAC-601AP; FabGennix), AC7 (PAC-701AP; FabGennix), ANP (sc-515701; Santa Cruz Biotechnology), BNP (sc-271185; Santa Cruz Biotechnology), GRK2 (sc-13143; Santa Cruz Biotechnology), *p*NF-κB (3033T; Cell Signaling Technology), NF-κB (8242T; Cell Signaling Technology), Cleaved Caspase-3 (9661T; Cell Signaling Technology), Collagen Type I (14695-1-AP, Proteintech), Collagen Type III (13548-1-AP, Proteintech), and GAPDH (10494-1-AP; Proteintech). Immunoblots were performed in triplicates and normalized with their respective loading controls.

### Statistical Analysis

One-way ANOVA was used for comparing data of multiple groups, and *post hoc* analyses were done using Tukey’s multiple comparisons test. Values of *p* < 0.05 were deemed significant. Using GraphPad Prism (Prism Version 8.0.2), all data were expressed as mean ± SEM.

## Results

### Chronic Catecholamine Stress Alters Cardiac and Inflammatory Functional Proteins in Mice

Analysis of mRNAs and protein expressions demonstrated that crucial cardiac and inflammatory proteins were altered in the myocardia during CCS compared with under physiological states. βARs, AC5, and AC7 were found depleting, while AC6, β-ARR-1, β-ARR-2, GRK2, and GRK5 were upregulated in the PCH mice hearts (Supplementary Figures 2A–K). Also, the TFs: GATA4, NFAT, MEF2, and NF-κB were overexpressed in PCH hearts compared with Ctrl and Vhl hearts (Supplementary Figures 3A–E). Cardiac hypertrophy was evident in PCH mice as atrial natriuretic peptide (ANP) and brain natriuretic peptide (BNP) were significantly upregulated in their myocardia than Ctrl and Vhl mice (Supplementary Figures 2A,J,K). To ascertain the impact of AC5, AC6, and AC7 alterations in PCH models, cAMP bioavailability was assessed and found to be significantly decreased in myocardial lysates obtained from PCH mice (Supplementary Figure 2L).

### Troponin and Lipopolysaccharide Elicit Similar Immunogenic Response During Chronic Catecholamine Stress

**FIGURE 1:**
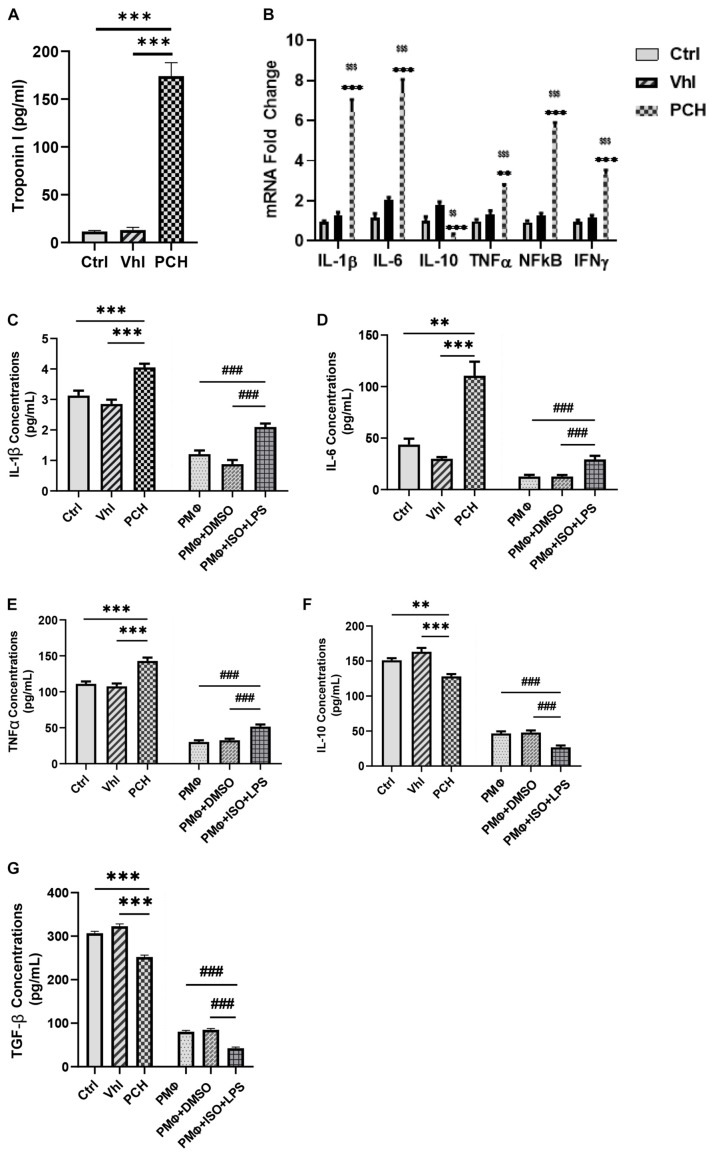
Troponin from necrotic cardiomyocytes and lipopolysaccharides (LPS) elicits similar inflammatory responses during chronic catecholamine stress (CCS). **(A)** Sera concentrations of troponin I were evaluated by ELISA from Control (Ctrl), Vehicle (Vhl), and Pathological cardiac hypertrophy (PCH) groups. ^∗∗∗^*p* < 0.001 **(B)** mRNA expressions of inflammatory markers (IL-1β, IL-6, TNFα, IFNγ, and NF-κB) assessed from the myocardial by qRT-PCR (*n* = 6 hearts per treatment group). ^∗∗^*p* < 0.01, ^∗∗∗^*p* < 0.001 vs. Vhl; ^$$^*p* < 0.01, ^$$$^*p* < 0.001 vs. Ctrl. **(C–G)** Comparison with inflammatory cytokines secretion (IL-1β, IL-6, TNFα, IL-10, and TGF-β) between PCH mice (*in vivo*) and LPS-challenged peritoneal macrophages (PMϕ) (*in vitro*) during CCS. All ELISA evaluations were performed in triplicates (*n* = 8 mice per treatment group). ^∗∗^*p* < 0.01, ^∗∗∗^*p* < 0.001 among *in vivo* groups; ^###^*p* < 0.001 among *in vitro* groups. Data are expressed as mean ± SEM. Data were analyzed using one-way ANOVA, followed by Tukey’s *post hoc* analysis. Abbreviations: IL, interleukin; TNFa, tumor necrosis factor-alpha; TGF-β, transforming growth factor-beta; IFNy, interferon-gamma.

Immediate assays for cardiac troponin I (cTnI) from sera showed its overt upregulation in PCH mice compared with concentrations in Ctrl and Vhl mice (Figure 1A). Assessment of mRNAs of IL-1β, IL-6, TNFα, IFNγ, NF-κB, and IL-10 from the myocardia showed that besides the latter, the proinflammatory cytokines and TF were significant overexpression in PCH hearts (Figure 1B).

By using LPS to mimic the immunogenicity of troponin *in vitro*, inflammatory responses elicited under stress were ascertained and compared with the *in vivo* models. The result demonstrated similarities in their immunogenicity. The PM_ϕ_ culture with ISO and LPS for 24 h secreted enormous amounts of IL-1β, IL-6, and TNFα just as observed in PCH mice, while IL-10 and TGF-β were also dampened similarly, both *in vitro* and *in vivo* (Figures 1C–G).

### Amlexanox and Forskolin Combination Inhibits Proinflammatory Responses During Chronic Catecholamine Stress Primarily *via* Cyclic Adenosine Monophosphate-Mediated Immunoregulation

**FIGURE 2:**
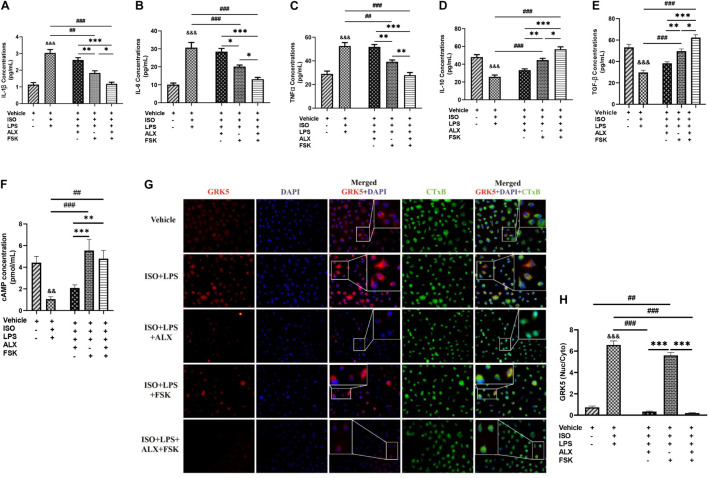
Amlexanox (ALX) and forskolin (FSK) combination inhibits PM_ϕ_ proinflammatory responses elicited during CCS, mostly *via* cyclic adenosine monophosphate (cAMP)-mediated immunoregulation. **(A-E)** ELISA evaluation of inflammatory cytokines (IL-1β, IL-6, TNFα, IL-10, and TGF-β) secreted by LPS-challenged PM_ϕ_ after employing ALX and/or FSK treatment intervention during CCS. **(F)** cAMP concentrations were assessed to determine the impact of the treatment interventions on ensuring cAMP bioavailability during CCS. The therapeutic groups are ALX treatment, FSK treatment, and ALX and FSK combination treatment. All ELISA evaluations were performed in triplicates (*n* = 8 mice per treatment group). ^∗^*p* < 0.05, ^∗∗^*p* < 0.01, ^∗∗∗^*p* < 0.001 among the therapeutic groups; ^##^*p* < 0.01, ^###^*p* < 0.001 LPS + PM_ϕ_ + ISO vs. therapeutic groups; ^&&^*p* < 0.01, ^&&&^*p* < 0.001 vs. LPS + PM_ϕ_ + Vhl. **(G)** Representative immunofluorescence of G protein-coupled receptor kinase 5 (GRK5) localizations, nuclei (DAPI), and cytoplasmic membrane (CTxB). **(H)** The plotted values are the GRK5 (nuclear/cytoplasm) expression ratios assessed from each PM_ϕ_ (*n* = 12–15 cells per four mice per treatment group). Color channels were adjusted in the merged images to enhance the visualization of all the respective fluorescence dyes. ^∗∗∗^*p* < 0.001 among the therapeutic groups; ^##^*p* < 0.01, ^###^*p* < 0.001, LPS + PM_ϕ_ + ISO vs. therapeutic groups; ^&&&^*p* < 0.001 vs. LPS + PM_ϕ_ + ISO. Data are expressed as mean ± SEM. Data were analyzed using one-way ANOVA, followed by Tukey’s *post hoc* analysis.

ALX (35 μM/ml) and/or FSK (10 μM/ml) treatments were employed to attenuate the hyperactive proinflammatory cytokine secretions elicited from the PM_ϕ_ *in vitro*. Analysis of the concentrations of inflammatory cytokines from these experiments using ELISA demonstrated that ALX treatment could not effectively subdue the hypersecretions of IL-1β, IL-6, and TNFα; neither did it upregulate IL-10 and TGF-β secretions during stress. Comparatively, FSK treatment was effective than ALX treatment at decreasing the proinflammatory responses while upregulating IL-10 and TGF-β secretions (Figures 2A–E). Regardless, ALX and FSK combination treatment was the most potent in subduing the hyperactive proinflammatory response.

Assessment of the effect of FSK on ensuring cAMP bioavailability showed that its presence in both the single and combination treatments upregulated cAMP markedly compared with the ISO + LPS and ISO + LPS + ALX groups (Figure 2F). Hence, it is inferred that the potency attained by the combination treatment may have been mainly *via* cAMP-mediated immunoregulation. The validation of this was done by assessing the effect of the therapies on GRK5 expressions. GRK5 cytosolic and nuclear expressions were inhibited by ALX in both the single and combination treatments, while FSK treatment had increased GRK5 nucleic–cytosolic expression ratio just as ISO + LPS, which had no treatment interventions (Figures 2G,H). However, proinflammatory responses were minimized in the ISO + LPS + FSK (Figures 2A–C), despite the increase in GRK5 nuclear expression.

### Amlexanox and Forskolin Combination Attenuates Left Ventricular Systolic Dysfunction in Mice During Chronic Catecholamine Stress

**FIGURE 3:**
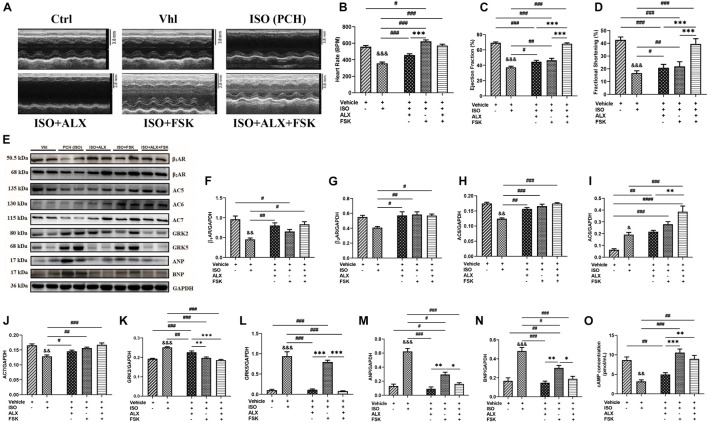
ALX and FSK combination attenuates left ventricular systolic dysfunction (LVSD) in mice during CCS by normalizing protein expressions. **(A)** Representative short-axis view M-mode echocardiogram imaging from Ctrl, Vhl, isoproterenol (ISO), ISO + ALX, ISO + FSK, and ISO + ALX + FSK. **(B**–**D)** Graphical presentations of heart rates, ejection fraction, and fractional shortening assessments from all models. The Vhl mice group is representative of the Ctrl mice group in the graphical presentations due to similarity in their data. The therapeutic groups are ALX treatment, FSK treatment, and ALX and FSK combination treatment (*n* = 10–11 hearts per treatment group). **(E**–**N)** Representative Western blots and graphical presentation of βARs, ACs, GRKs, ANP, and BNP, were assessed from all groups (*n* = 4 hearts per treatment group). Western blots were performed in triplicates, and each protein band in the representative blot is an independent biological sample. **(O)** Graphical represents of cAMP concentrations evaluated by ELISA (*n* = 9 mice per treatment group). ^∗^*p* < 0.05, ^∗∗^*p* < 0.01, ^∗∗∗^*p* < 0.001 among the therapeutic groups; &&*p* < 0.01, ^&&&^*p* < 0.001 vs. Vhl; ^#^*p* < 0.05, ^##^*p* < 0.01, ^###^*p* < 0.001 vs. the therapeutic groups. Data are expressed as mean ± SEM. Data were analyzed using one-way ANOVA, followed by Tukey’s *post hoc* analysis. Abbreviations: βARs, β-adrenergic receptors; ACs, adenylyl cyclases; GRKs, G-protein-coupled receptor kinases; ANP, atrial natriuretic peptide; BNP, brain natriuretic peptide.

Echocardiography and ECG assessments at the end of *in vivo* model showed that PCH mice had LVSD. The treatments of ALX, FSK, and their combination were employed during CCS in attempts to prevent the LVSD. Cardiac functions assessed after using these treatment interventions showed that ALX failed to prevent the LVSD during CCS, as ejection fraction (EF) and fractional shortening (FS) were still decreased significantly compared with the control groups. Similarly, FSK treatment did not attenuate the LVSD during CCS but instead caused tachycardia and arrhythmias (Figures 3A–D and Supplementary Figure 3F and Supplementary Table 2). Intriguingly, ALX and FSK combination was found to preserve cardiac function by maintaining EF and FS during CCS.

### Amlexanox and Forskolin Combination Normalizes Cardiac and Inflammatory Functional Protein Expression in Mice During Chronic Catecholamine Stress

To ascertain the potential mechanism of action employed by the ALX and FSK combination to prevent the LVSD, the expression of functional proteins and TFs were reassessed. βARs, AC5, AC6, AC7, GRK2, GRK5, ANP, BNP, GATA4, NFAT, MEF2, and NF-κB expressions were found to have been altered in PCH mice myocardia (Supplementary Figures 2, 3). However, the ALX and FSK combination prevented significant alteration in the expressions of these proteins, while their individual treatment showed several limitations. β_1_AR and β_2_AR expressions were sustained by all treatment intervention groups. FSK in both single and combination treatment normalized AC5 and AC7 to physiological levels, while AC6 was upregulated; thus, cAMP concentrations were primarily increased in these groups. Meanwhile, ALX showed a negligible inhibitory effect on GRK2 but prevented GRK5, ANP, and BNP upregulations in both its single and combined treatments with FSK (Figures 3E–O). Nonetheless, GATA4, NFAT, MEF2, and NF-κB expressions were still upregulated predominantly in the ALX treatment group and followed by the FSK treatment group. Intriguingly, ALX and FSK combination minimized the expressions of these functional proteins and TFs close to physiological levels during CCS (Supplementary Figures 4A–E).

### Amlexanox and Forskolin Combination Preserves Mice Myocardial Architecture During Chronic Catecholamine Stress

**FIGURE 4:**
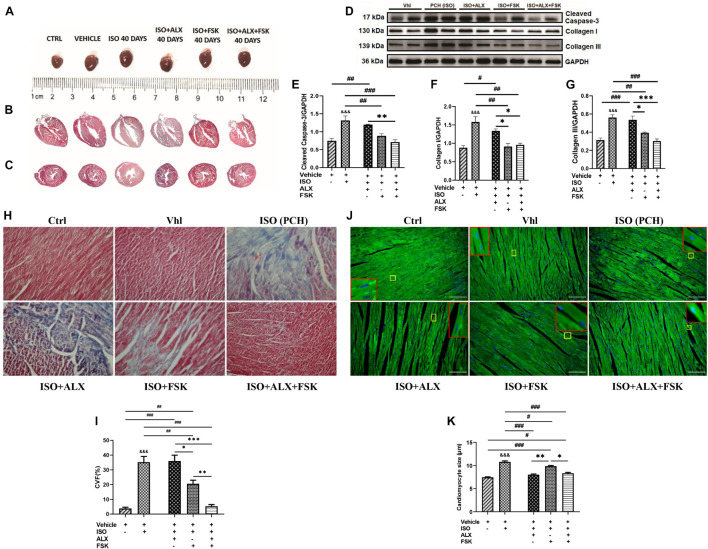
ALX and FSK combination preserves mice myocardial architecture during CCS. **(A)** Representative whole hearts from all experimental groups. **(B)** Representative Masson’s trichrome stained longitudinal section of whole hearts from all groups. **(C)** Representative Masson’s trichrome stained transverse section of whole hearts from all groups. **(D–G)** Representative Western blots and graphical presentations cleaved caspase 3, collagen I, and collagen III compared between Vhl and PCH mice, and ALX treatment, FSK treatment, and ALX and FSK combination treatment (*n* = 4 hearts per treatment group). Western blots were performed in triplicates, and each protein band in the representative blot is an independent biological sample. **(H,I)** Representative microscopic images of Masson’s trichrome staining and collagen volume fraction (CVF) in the ventricular tissue sections from all groups. The plotted values are the means of the CVF from each mouse (*n* = 6–8 fields of view per 5–7 sections per 8–16 hearts per group). **(J,K)** Representative microscopic images of wheat germ agglutinin (WGA) merged with DAPI staining and graphical presentation of measured cardiomyocyte diameters from ventricular tissue sectionings across all groups. The plotted values are the means of the cardiomyocyte sizes from each mouse (*n* = 10–12 cells per five fields of view per five sections per six to seven hearts per group). Representative cardiomyocytes are marked in yellow boxes, and their zoomed-in (× 5) inserts to show hypertrophy are marked in red boxes. The Vhl mice group is representative of the Ctrl mice group in results illustrations due to similarity in their data. ^∗^*p* < 0.05, ^∗∗^*p* < 0.01, ^∗∗∗^*p* < 0.001 among the therapeutic groups; ^&&&^*p* < 0.001 vs. Vhl; ^#^*p* < 0.05, ^##^*p* < 0.01, ^###^*p* < 0.001 vs. the therapeutic groups. CVF was estimated by dividing the collagen area with the total myocardial area and multiple by 100. Data are expressed as mean ± SEM. Data were analyzed using one-way ANOVA, followed by Tukey’s *post hoc* analysis.

LV posterior wall thickness (LVPW), LV mass (LVM), and heart weight/body weight (HW/BW) ratio assessments showed their significant increases in PCH mice compared with the Vhl and Ctrl groups (Figure 4A and Supplementary Table 2). However, during CCS, ALX in both single and combination treatment prevented significant increases in LVPW, LVM, and HW/BW; meanwhile, FSK treatment failed to achieve these. Masson’s trichrome staining of longitudinal and transverse myocardial sections showed apparent distortions of the gross cardiac morphological of PCH mice. Nonetheless, rather than either ALX or FSK treatments, their combination seemed to have preserved the myocardial architecture during CCS (Figures 4B,C). The impacts of the treatment interventions on cardiomyocyte apoptosis and collagen depositions were ascertained to validate this observation. Cleaved caspase-3 (biomarker for apoptosis) was upregulated in PCH hearts along with significant increases in collagen I and III. In conformity with the histological findings, only the ALX and FSK combination treatment was potent at preventing cardiomyocyte apoptosis and collagen depositions in the heart of the mice during CCS (Figures 4D–G). Also, the myocardial collagen volume fraction (CVF) assessed further confirmed that PCH mice had marked interstitial deposits, which was only prevented effectively by the ALX and FSK combination treatment (Figures 4H,I). Besides, cardiomyocyte diameters measured from WGA and H&E staining showed excessive myocyte hypertrophy in PCH hearts. Consistent with attenuating the increases in ANP, BNP, LVPW, HW/BW, and LVM, ALX in both single and combination treatment prevented cardiomyocyte hypertrophy during CCS, while FSK treatment could not (Figures 4J,K and Supplementary Figures 4F,G).

### Amlexanox and Forskolin Combination Prevents Proinflammatory Response Aggravation in Mice Myocardia During Chronic Catecholamine Stress

**FIGURE 5:**
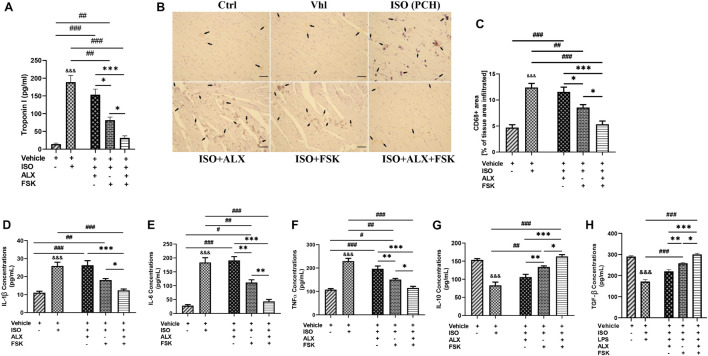
ALX and FSK combination prevents proinflammatory responses aggravation in myocardia during CCS by preventing necrosis. **(A)** Graphical presentations of evaluated sera concentrations of troponin I from groups. The sera troponin I analysis was performed in triplicates (*n* = 8 mice per treatment group). ^∗^*p* < 0.05, ^∗∗∗^*p* < 0.001, among the therapeutic groups; ^&&&^*p* < 0.001 vs. ISO (PCH); ^##^*p* < 0.01, ^###^*p* < 0.001, vs. the therapeutic groups. **(B,C)** Representative CD68 IHC staining and graphical presentation of the extent of inflammatory cells infiltration into the myocardial of all groups. The Vhl mice group is representative of the Ctrl mice group in results illustrations due to similarity in their data (*n* = 6–8 field of view per six to eight sections per six to seven hearts per group). **(D–H)** Graphical presentations of inflammatory cytokines (IL-1β, IL-6, TNFα, IL-10, and TGF-β) were evaluated by ELISA from all groups. The cytokines analysis was performed in triplicates (*n* = 7–8 mice per treatment group). ^∗^*p* < 0.05, ^∗∗^*p* < 0.01, ^∗∗∗^*p* < 0.001, among the therapeutic groups; ^&&&^*p* < 0.001 vs. Vhl; ^#^*p* < 0.05, ^##^*p* < 0.01, ^###^*p* < 0.001 vs. the therapeutic groups. Data are expressed as mean ± SEM. Data were analyzed using one-way ANOVA, followed by Tukey’s *post hoc* analysis.

The effects of the treatment interventions in modulating myocardial inflammation were initially ascertained by evaluating their impact on cardiomyocyte necrosis through the immediate sera analysis of the cTnI. Inference from the cTnI concentrations indicated that the ALX treatment was ineffective at preventing necrosis, while FSK treatment did better comparatively. Regardless, ALX and FSK combination was the most potent in preventing necrosis during CCS (Figure 5A). Furthermore, CD68^+^ IHC staining was done to evaluate the efficacies of the treatments in attenuating the aggravated inflammatory cell infiltration observation in PCH hearts (Figures 5B,C) and demonstrated that, while FSK significantly decreased the CD68^+^ cells infiltration than the ALX during CCS, their combination was the most potent at preventing exacerbation of the inflammatory responses. In conformity, the followed-up cytokine analysis showed that the ALX and FSK combination was the most effective in downregulating IL-1β, IL-6, and TNFα secretions while enhancing IL-10 and TGF-β. Comparatively, FSK treatment was able to do these better than ALX treatment did during CCS (Figures 5D–H).

## Discussion

**FIGURE 6:**
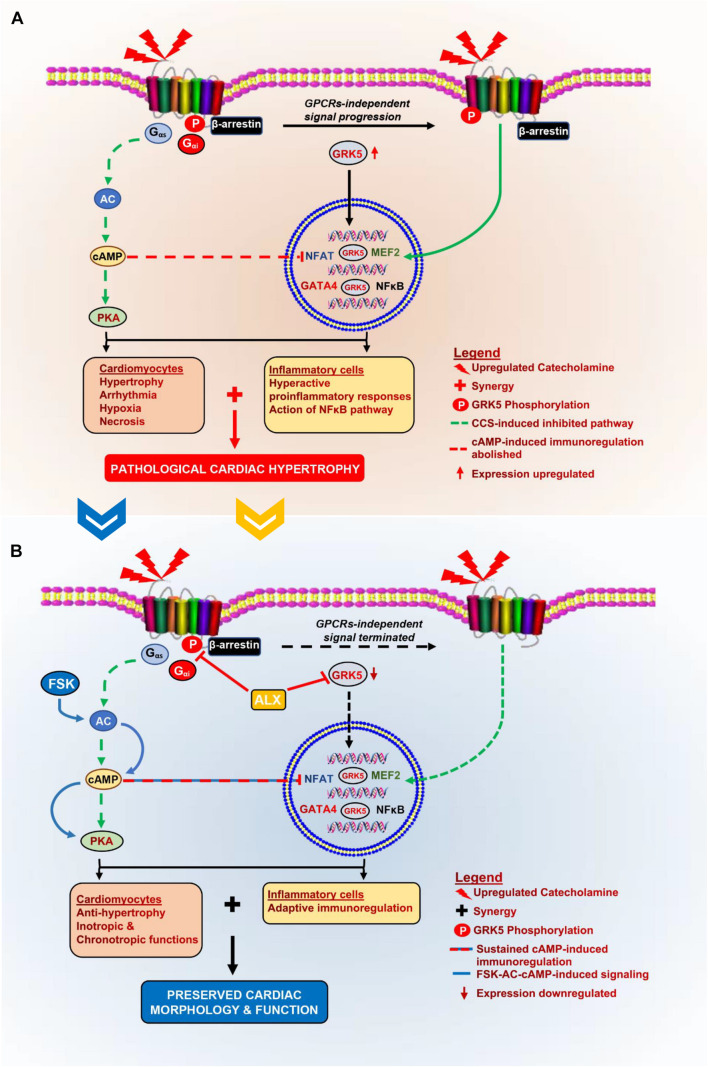
Schematics of the pathomechanism of isoproterenol-induced cardiomyopathy and the mechanisms of ALX and FSK combination treatment. **(A)** During CCS, G_αs_-AC is decoupled from βARs, thereby inhibiting cAMP synthesis, which affects cardiac function and immunoregulation in the myocardia adversely. Also, GRK5 upregulates and phosphorylates βARs as well as translocations into the nuclei to induce GPCRs-independent stimuli signaling. The nuclear activity of GRK5 induces the activation of cardiomyocyte hypertrophy transcriptional factors. These result in excessive cardiomyocyte hypertrophy and hyperactive proinflammatory responses. At the same time, cAMP-mediated adaptive immunoregulation is abolished. The synergy of these cascades results in the exacerbation of collagen deposits which pathologically remodels the heart. **(B)** The administration of ALX and FSK combination facilitates ALX-GRK5 inhibition (expressions and nuclear translocation), which prevents cardiomyocyte hypertrophy; while, FSK-ACs-cAMP modulates adaptive immunoregulation and cardiac inotropic functions. Combining these mechanisms preserves cardiac morphology and prevents left ventricular systolic dysfunction (LVSD) during CCS.

This study aimed to prevent isoproterenol-induced cardiomyopathy through the attenuation of maladaptive myocardial hypertrophy and inflammatory responses. The preliminary assessment of cardiac and inflammatory functional proteins demonstrated their alteration during CCS compared with physiological states. Typically, β_1_AR expressions were significantly downregulated, while β_2_AR expressions were rarely depleted during CCS. Consistent with previous studies, β_2_AR is implicated in mediating stress-induced cardiovascular diseases (Paur et al., 2012; Spadari et al., 2018). Also, while AC5 expression was decreased during CCS, AC6 was found upregulated. In conformity with these findings, it has been previously shown that AC5 facilitates PKA pathway signaling under physiological conditions, while AC6 mediates stress response for calcium channel modulation *via* PKA/STAT3 (Wu et al., 2017). Like AC5, AC7 was downregulated in PCH mice myocardia compared with the Ctrl and Vhl groups. The ELISA, performed to ascertain the impact of the ACs alteration on cAMP bioavailability, showed decreased concentrations in PCH mice as reported in other chronic stress models (Plattner et al., 2015). cAMP mediates crucial cardiac and immunoregulatory functions *via* the modulation of the LTCC, NFAT, MEF2, and NF-κB in both inflammatory and cardiac cells (Pereira et al., 2015; Raker et al., 2016). Hence, the observed downregulation of cAMP in PCH mice might permit cardiac and inflammatory dysfunctions. We also found that β-ARR-1, β-ARR-2, GRK2, and GRK5 were overexpressed in PCH hearts, which were consistent with the findings of others (Hullmann et al., 2014; Adzika et al., 2019; Sun et al., 2021). GRK2 upregulation increases the internalization of β_1_AR *via* the recruitment of β-ARR-1 in the homologous desensitization process (Adzika et al., 2019; Sun et al., 2021); therefore, this explains the downregulated expression of β_1_AR in PCH myocardia. Conversely, by recruiting β-ARR-2, GRK5 enhances β_2_AR signal progression in a GPCR-independent manner to induce maladaptive hypertrophy and proinflammatory responses *via* phosphorylating NFAT, MEF2, GATA4, and NF-κB (Patial et al., 2011; Islam et al., 2013; Hullmann et al., 2014; Figure 6). Intriguingly, these cardiac and inflammatory TFs were shown overexpressed, along with ANP and BNP upregulated in the PCH myocardia compared with their expressions in the control groups (Supplementary Figures 3, 4). These are indicative of the likelihood of GRK5 activities in inflammatory and cardiac cells.

The exacerbation of cardiovascular remodeling by cTnI-induced inflammatory responses has also been widely reported. In these instances, elevated proinflammatory and downregulated anti-inflammatory responses were observed (Kaya et al., 2008; Heidt et al., 2014). The upregulated sera levels of cTnI in PCH mice (Figure 1A) indicate the occurrence of excessive cardiomyocyte necrosis. In line with the increased release of cTnI by necrotic cardiomyocytes, proinflammatory responses (IL-1β, IL-6, TNFα, IFNγ, and NF-κB) were hyperactive in PCH mice, while anti-inflammatory responses (IL-10 and TGF-β) were dampened, just as demonstrated previously (Kaya et al., 2008).

Furthermore, LPS has been extensively used to induce cardiac inflammation and has been observed to share similarities in the immunogenic responses elicited by troponin (Cai et al., 2020). To validate this, we mimicked cTnI immunogenicity in PCH models with LPS under stress conditions *in vitro*. Comparing cytokine analysis between *in vivo* and *in vitro* experiments confirmed the similarities between cTnI and LPS immunogenicity during stress. Also, although it could be argued that LPS would still elicit proinflammatory responses from PM_ϕ_ under physiological state, a previous study and performed cytokine analysis (data not included) have demonstrated the exacerbation of these responses during CCS (Laukova et al., 2018).

In further *in vitro* investigations, we explored the inhibitory effects of ALX on GRK5 and/or the stimulatory effects of FSK on AC/cAMP synthesis for attenuating the LPS-induced inflammatory responses during stress. The followed-up cytokine analysis demonstrated that only the inhibition of GRK5 during stress could not effectively attenuate the proinflammatory response elicited from the PM_ϕ_, although the ALX dosage used was within the range reported previously to halt proinflammatory response in a different pathological model (Quan et al., 2019). Comparatively, FSK-AC/cAMP exerted more anti-inflammatory effects than ALX did; regardless, their combination showed the most potency at exhibiting adaptive immunoregulation. FSK enhanced cAMP bioavailability in single and combined treatments. Consistent with these findings, FSK-AC/cAMP had been reported earlier to exert anti-inflammatory effects *via* NF-κB inhibition (Chiadak et al., 2016). To validate the mechanism utilized by the ALX and FSK combination to attenuate the proinflammatory responses, immunofluorescence was used to ascertain the expressions of GRK5 among the *in vitro* treatment groups (Figures 2G,H). As suggested (Homan et al., 2014), ALX in both single and combination treatment inhibited GRK5 expression and nuclear translocation, while FSK failed to do either, just like the group without treatment interventions (LPS + ISO). However, although GRK5 translocated into the nuclei of LPS + ISO as much as it did in LPS + ISO + FSK, proinflammatory responses were not aggravated in the latter. As such, these were concluded from Figure 2 and the findings of others: (1) Although ALX inhibited GRK5-mediated proinflammatory response activation, other inflammatory mediators such as kinases, peptides, and amines are still capable of inducing the immune responses (Abdulkhaleq et al., 2018), as observed in the LPS + ISO + ALX group. (2) Also, the abolishment of cAMP-dependent adaptive immunoregulation by stress in the LPS + ISO + ALX group may have enabled the observed proinflammatory responses, as demonstrated previously (Bopp et al., 2009; Wehbi and Taskén, 2016). (3) Besides, unlike ALX, which attenuates GRK5-mediated inflammatory responses, FSK-ACs/cAMP adaptively regulates multiple anti-inflammatory cascades (Raker et al., 2016; Wehbi and Taskén, 2016). Hence, in line with previous studies (Bopp et al., 2009; Patial et al., 2011; Chiadak et al., 2016; Raker et al., 2016; Wehbi and Taskén, 2016), we confirmed that ALX and FSK combination attained their potency primarily *via* FSK/ACs/cAMP-mediated adaptive immunoregulation, coupled with ALX inhibiting the activation of maladaptive inflammatory responses *via* GRK5. Based on these findings, it was hypothesized that rather than either FSK or ALX treatment, their combination might attenuate myocardial inflammation, which drives the adverse remodeling of hearts during CCS.

These hypotheses were tested by translating ALX and/or FSK treatments *in vivo* during CCS. At the end of all models, echocardiogram results revealed that the ALX and FSK combination prevent LVSD during CCS, by maintaining typical heart rates, ejection fractions above 65%, and fraction shortenings above 35%. However, ALX treatment failed to maintain proper cardiac function during CCS, while FSK treatment resulted in arrhythmias. Contrary to our findings, Mo et al. (2020) reported that ALX improved cardiac function, but this was in acute myocardial infarction animal models and not during CCS or in an isoproterenol-induced cardiomyopathy model. Also, the ALX dosage they employed was 10 times more than what we used in this study. Nonetheless, our findings regarding FSK-induced arrhythmias were in conformity with the reports from previous studies (Christ et al., 2014). Intriguingly, tachyarrhythmia did not occur in the combined treatment group despite the presence of FSK, and we speculated that this might be due to an effect exerted by ALX.

Initially, in this study, the occurrence of LVSD was associated with alterations in cardiac and inflammatory proteins in the myocardia during CCS. As such, to confirm that the treatment interventions influenced their expression to preserve cardiac function, these proteins were reevaluated. In summary, the immunoblotting results showed that the ALX and FSK combination had normalized the expression of all the assessed cardiac and inflammatory proteins besides AC6, which is specifically responsible for cardiomyocyte protection during stress (Wu et al., 2017).

Although ALX treatment inhibited GRK5, the TFs, GATA4, NFAT, MEF2, and NF-κB were still overexpressed during CCS (Supplementary Figure 4). Consistent with these findings, Hullmann et al. (2014) had earlier suggested that the inhibition of GRK5 might not halt the activation of NFAT, which might have applied to GATA4, MEF2, and NF-κB as well. Contrarily, FSK single treatment decreased the expression of these TFs, just as Zoccarato et al. (2015) and Chiadak et al. (2016) had demonstrated.

Also, the myocardial morphometric data indicated that the hearts of PCH mice were hypertrophied and had marked fibrosis, which were determined with measured cardiomyocyte diameters, upregulated ANP and BNP upregulation, and Masson’s-stained tissues. Further investigations with cleaved caspase-3 and collagen I and III immunoblotting confirmed a significant increase in cardiomyocyte apoptosis and collagen depositions in PCH myocardial. Intriguingly, ALX and FSK combination attenuated excessive cardiomyocyte hypertrophy, apoptosis, and myocardial fibrosis during CCS. Meanwhile, neither ALX nor FSK treatments were able to achieve all the aforementioned individually. ALX treatment downregulated ANP and BNP and attenuated GRK5-induced hypertrophy as suggested (Homan et al., 2014), but it failed to prevent apoptosis, hence, the occurrence of fibrosis. Consistent with these findings, apoptosis has been well demonstrated to aggravate collagen deposition and fibrosis (Hinz and Lagares, 2020). In contrast to ALX treatment, FSK-AC-cAMP significantly inhibited cardiomyocyte apoptosis and fibrosis, just as demonstrated previously (Kwon et al., 2004; Lee et al., 2010; Younce et al., 2013). However, myocardial hypertrophy remained incompletely resolved by FSK treatment. Wolf et al. (2005) had previously reported that myocardial hypertrophy increases tachyarrhythmia susceptibility. Hence, this may have accounted for the observed tachyarrhythmia in the FSK treatment group but not in the ALX and FSK combination group since ALX had attenuated hypertrophy.

Lastly, assessing the effects of the treatment intervention on modulating myocardial inflammation during CCS showed that ALX and FSK combination prevents cardiomyocyte necrosis as sera concentrations of cTnI in mice from this group were significantly downregulated. Hence, cTnI-induced inflammation was attenuated in the ALX and FSK combination treatment group. Individually, in contrast with ALX, FSK-AC-cAMP significantly decreased cTnI (cardiomyocyte necrosis) during CSS, as demonstrated in other studies (Wang et al., 2016). Furthermore, in correlation with the extent of necrosis shown in PCH hearts, enormous amounts of CD68^+^ inflammatory cells were found to have infiltrated their myocardia. This provides supporting evidence that myocardial inflammation is exacerbated by stress, as demonstrated previously (Scally et al., 2019).

Nonetheless, we found that the ALX and FSK combination treatment was the most potent in minimizing CD68^+^ inflammatory cell infiltration into the myocardial during CCS, although FSK treatment also attained this significantly. Similarly, FSK-AC/cAMP has been shown to adaptively modulate myocardial inflammatory responses (Lee et al., 2010; Shim et al., 2014). However, regarding ALX treatment, we found that it was not effective at decreasing myocardial inflammation, as Mo et al. (2020) had reported. The discrepancies in these findings may be due to the differences in the cardiovascular disease model being investigated and the variation in ALX dosage employed. To complement our findings, the evaluated cytokine concentrates depicted ALX and FSK combination treatment during CCS as the most effective intervention at keeping the gap between proinflammatory (IL-1β, IL-6, and TNFα) and anti-inflammatory (IL-10 and TGF-β) responses close to a homeostatic immune state. This certainly contributed to the prevention of biased prolonged proinflammatory responses, which could have exacerbated myocyte necrosis, apoptosis, and aggravated interstitial collagen deposits, as shown in other studies (Xiao et al., 2018; Sandstedt et al., 2019).

## Conclusion

Here, we demonstrated the implication of maladaptive inflammatory response in the pathogenesis of isoproterenol-induced cardiomyopathy. Our findings showed that besides ALX preventing the occurrence of cardiomyocyte hypertrophy, it complemented the efforts of FSK-ACs/cAMP-mediated immunoregulation by abolishing GRK5-mediated induction of inflammatory responses. These combined efforts helped ALX and FSK maintain the myocardial homeostasis, thereby preserving the cardiac morphology and function during CCS (Figure 6). Owing to the clinical significance of this study, it is appropriate to acknowledge its limitations. It could be argued that ALX can inhibit IkB kinases and TANK-binding kinase 1 (TBK1) besides GRK5. Nonetheless, the inhibition of IkB kinases and TBK1 along with GRK5 by ALX, as demonstrated (Mo et al., 2020), still did not prevent the LVSD in PCH mice models. Also, further studies into determining the toxicity of ALX and FSK combined treatment and its impact on diastolic function and lung congestion during CCS will be required to fully ascertain their therapeutic and translational potentials.

## Data Availability Statement

The original contributions presented in the study are included in the article/Supplementary Material, further inquiries can be directed to the corresponding author/s.

## Ethics Statement

The animal study was reviewed and approved by the Experimental Animal Centre of Xuzhou Medical University and the Animal Ethics Committee of the Medical University.

## Author Contributions

GKA conceived the experiments ideas. HS and GKA designed the experiments. GKA and HH isolated and cultured PM_ϕ_. AA provided the experimental animals. RR assisted in making the animal models. SA and SK performed PM_ϕ_ identification. KL, Q-MD, and XM performed the cardiac function assessments. GKA, TM, and JA-A performed the sera cytokine analysis. GKA, SA, Q-MD, RM, and WS analyzed and interpreted the data. GKA, RM, MN, JA-A, JM, and WS drafted and wrote the manuscript with contributions from all authors. All authors discussed, proofread, and approved the manuscript.

## Conflict of Interest

The authors declare that the research was conducted in the absence of any commercial or financial relationships that could be construed as a potential conflict of interest.

## Publisher’s Note

All claims expressed in this article are solely those of the authors and do not necessarily represent those of their affiliated organizations, or those of the publisher, the editors and the reviewers. Any product that may be evaluated in this article, or claim that may be made by its manufacturer, is not guaranteed or endorsed by the publisher.

## Abbreviations

AC: adenylyl cyclase
ALX: Amlexanox
cAMP: cyclic adenosine monophosphate
CCS: chronic catecholamine stress
cTnI: cardiac troponin I
FSK: Forskolin
GRK: G protein-coupled receptor kinases
ISO: isoproterenol
LPS: lipopolysaccharide
LVSD: left ventricular systolic dysfunction
PCH: pathological cardiac hypertrophy
PM_ϕ_: peritoneal macrophage
TBK1: TANK-binding kinase 1
TFs: transcriptional factors
TNFα: tumor necrosis factor-alpha
βAR: beta-adrenergic receptors.
