# Supplementary material for: Amlexanox and Forskolin Prevents Isoproterenol-Induced Cardiomyopathy by Subduing Cardiomyocyte Hypertrophy and Maladaptive Inflammatory Responses
Source: Front Cell Dev Biol. 2021 Sep 24;9:719351. doi: 10.3389/fcell.2021.719351 (PMC8497899; doi:10.3389/fcell.2021.719351)
Supplement: Supplementary file 1 [file Data_Sheet_1.docx]

**Figure S1: Experimental design for in vitro and in vivo experiments**

**Figure S2: CCS induces PCH by altering the expressions of cardiac and immune functional proteins. (A)** Gene expressions of G_αs_, AC5, AC7 were significantly upregulated in the PCH mice, while G_αi_ is rare depleted. Also, mRNAs of AC6, GRK2, GRK5, ANP, and BNP are significantly elevated in the PCH mice compared to their expressions in Ctrl and Vhl mice (n=6 hearts per each treatment group). ++p<0.01, +++p<0.001 vs Vhl. $$p<0.01, $$$p<0.001 vs Ctrl; £p<0.05. **(B-K)** Western blotting illustrated the alteration in the gene expressions were translated into their protein expressions (n=6 hearts mice per each treatment group). Western blots were performed in triplicates, and each protein band in the representative blot is an independent biological sample. *p<0.05, **p<0.01, ***p<0.001 **(L)** Graphical represents of cAMP concentrations evaluated by ELISA (n=9 mice per treatment group). **p<0.01. Data are expressed as mean ± SEM.

**Figure S3: CCS induces PCH by altering the expressions of cardiac and inflammatory transcriptional factors. (A-E)** Western blotting results demonstrate upregulation in GATA, NFAT, MEF2, and NF-κB in PCH mice compared to Ctrl and Vhl mice (n=6 hearts per treatment group). Western blots were performed in triplicates, and each protein band in the representative blot is an independent biological sample. (**F**) Representative electrocardiogram imaging from Ctrl, Vhl, ISO, ISO+ALX, ISO+FSK, and ISO+ALX+FSK displaying heart rate (Green), respiration rate (yellow) and temperature (turquoise). **p<0.01, ***p<0.001. Data are expressed as mean ± SEM.

**Figure S4: ALX and FSK combination normalizes the expressions of cardiac and inflammatory TFs during CCS. (A-E)** Representative western blots and graphical presentations of GATA4, NFAT, MEF2, and NF-κB protein expressions of all groups (n=4 hearts per treatment group). Western blots were performed in triplicates, and each protein band in the representative blot is an independent biological sample. **(F and G)** Representative microscopic images of H&E staining and measured cardiomyocyte diameters from ventricular tissue sectionings across all groups. The plotted values are the means of the cardiomyocyte sizes from each mouse (n=10-12cells per 5 field of view per 6-8 sections per 6-8 hearts per group). *p<0.05, **p<0.01, ***p<0.001 among the therapeutic groups; &&&p<0.001 vs ISO (PCH); # p<0.05, ##p<0.01, ###p<0.001 vs the therapeutic groups. Data are expressed as mean ± SEM. Data were analyzed using one-way ANOVA, followed by Tukey’s post hoc analysis.

**Fig. S1**


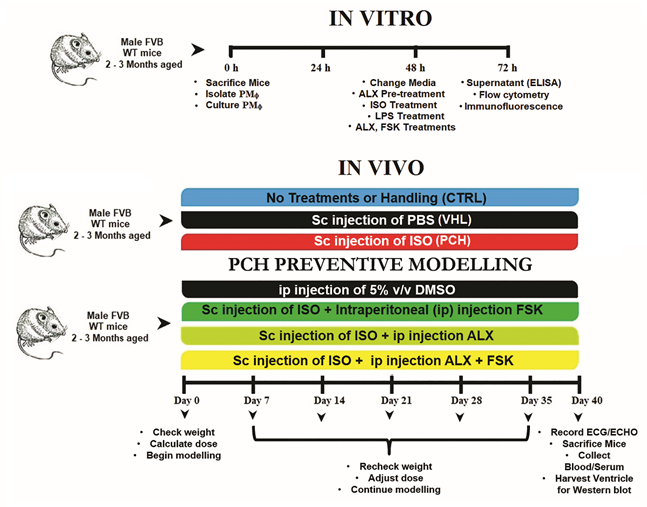


**Fig. S2**

**A**


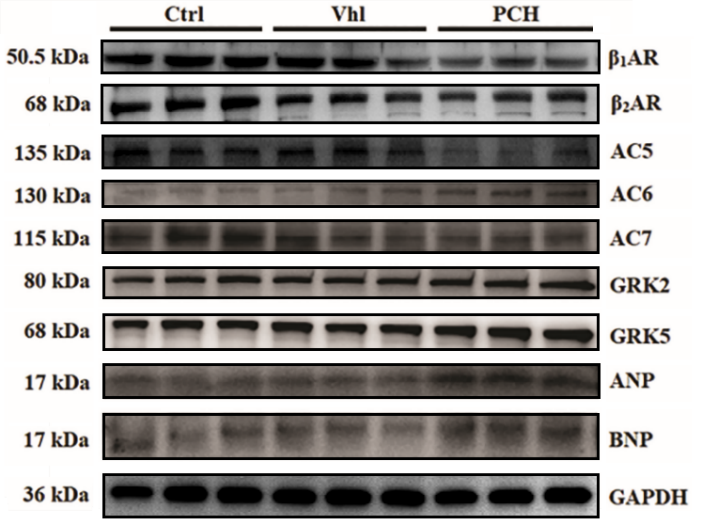


**C**

**B**

**E**

**D**


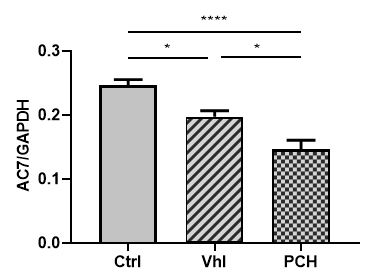


**L**

**K**

**J**

**I**

**H**

**G**

**F**

**Fig. S3**

**A**


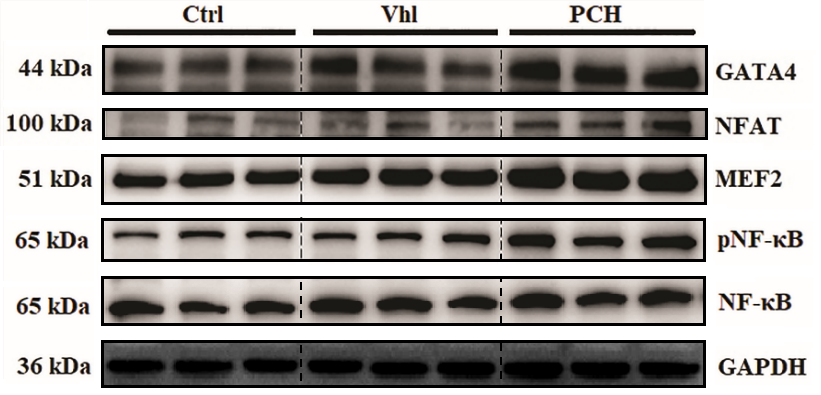


**F**

**E**

**C**

**D**

**B**

**Ctrl**

**Vhl**

**ISO(PCH)**

**ISO+ALX**

**ISO+FSK**

**ISO+ALX**

**+FSK**


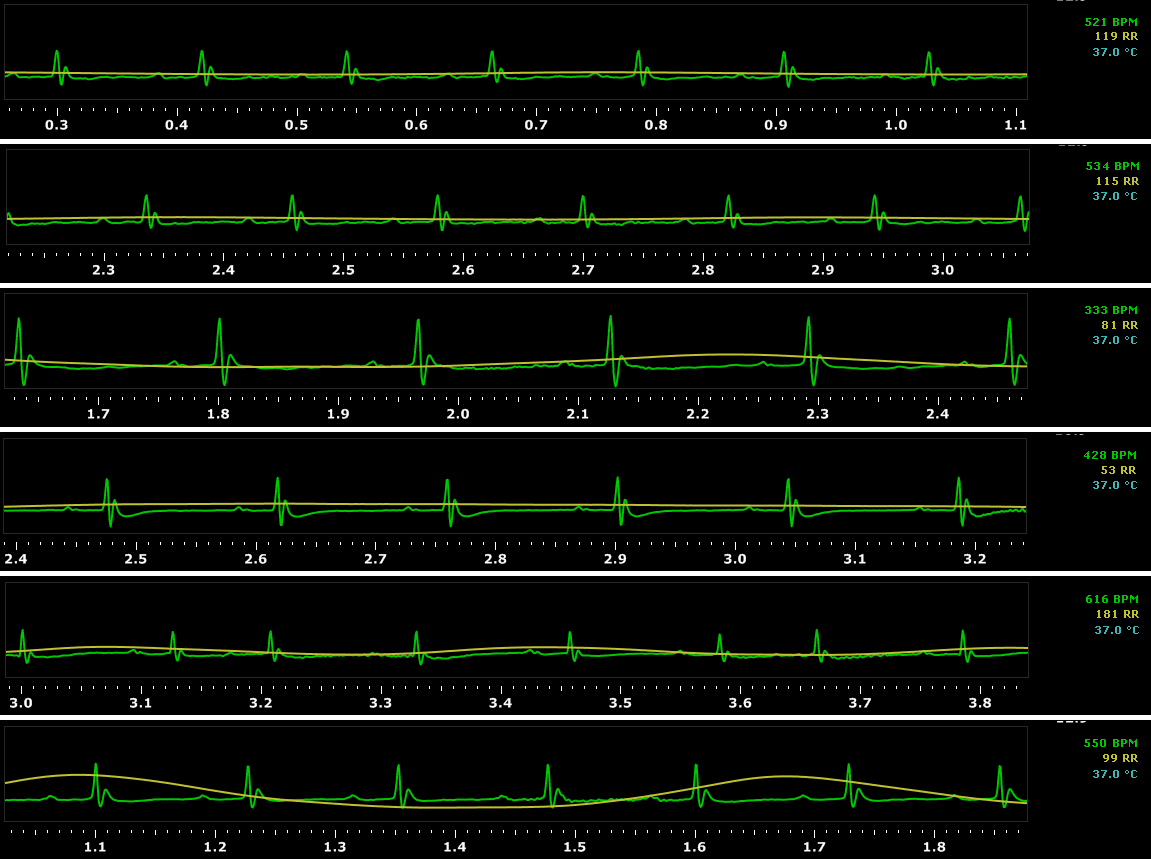


**Fig. S4**

**
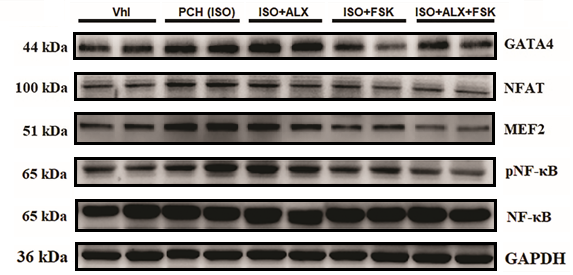
**

**C**

**D**

**E**

**B**

**A**

**F**

**Ctrl Vhl ISO (PCH)**

**
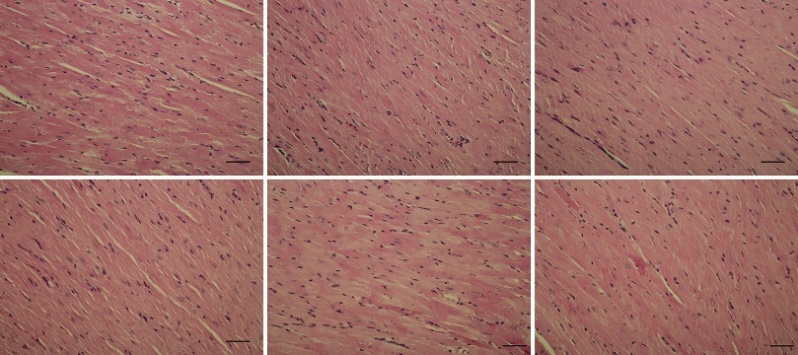
**

**G**

**ISO+ALX ISO+FSK ISO+ALX+FSK**

**Tables**

**Table S1: List of primer sequence used for RT-qPCR**

| **No.** | **Gene** | **Antisense** | **Sense** |
| --- | --- | --- | --- |
| 1 | AC5 | CAGAGCCAGCACGAAGACAGA | TCACCGCCAATGCCATAGACT |
| 2 | AC6 | AGAACCGAGAAGATTCCAACC | TTCCCATACTCCACCCTGATA |
| 3 | AC7 | GGAGCAGCCGTGTCCTTTGTG | TGTTTTCCTGCGGATGAGCCT |
| 4 | GRK2 (Adrbk1) | GGTTGGGGAACAAGTAGAAGT | GAGGACTATGCCCTGGGTAAG |
| 5 | GRK5 | CAAGATGTTTTCGGGTTTTAG | AAGAGCGAGCCTTATTTTATG |
| 6 | Gnai2 | TGACCACCCACATCAAACATC | ATGACTCAGCCGCTTACTACC |
| 7 | Gnas | ATCCTCAGGAGTGGTGTAGCG | TGTGACTGCCATCATCTTCGT |
| 8 | ANP | CTGCTTCGGGGGGTAGGATTGAC | TGACACACCACAAGGGCTTAGGA |
| 9 | BNP | CGAGACAAGGGAGAACAC | AAGGAAAAGCAGAAACAGAA |
| 10 | IL-1β | GTGTTTTCCTCCTTGCCTCTGAT | GCTGCCTAATGTCCCCTTGAAT |
| 11 | IL-6 | TCACAGAAGGAGTGGCTAAGGACC | ACGCACTAGGTTTGCCGAGTAGAT |
| 12 | IL-10 | GGAAGACAATAACTGCACCCACT | CAACCCAAGTAACCCTTAAAGTCC |
| 13 | TNFα | GAAAGCATGATCCGAGATGTG | CACGAGCAGGAATGAGAAGAG |
| 14 | IFNγ | AGTGGCATAGATGTGGAAGAAAAGA | TCAGGTGTGATTCAATGACGCTTAT |
| 15 | NF-kB | TCTGAACAAAATGCCCCACGGT | GCCTTCCTCCTTTGGGACGATG |
| 16 | β-ARR-1 | TTCTTGATGAGTCGCTCCTGT | TGTCTTGGGTCTGACTTTTCG |
| 17 | β-ARR-2 | GAATTGTTGGTGACATGGACG | GAGGAACTCTGTGCGGCTTAT |
| 18 | GAPDH | TGTGATGGGTGTGAACCACGAGAA | AGTGATGGCATGGACTGTGGTCAT |

| Parameter | Ctrl | Vhl | PCH (ISO) | ISO+ALX | ISO+FSK | ISO+ALX+FSK |  |
| --- | --- | --- | --- | --- | --- | --- | --- |
| Morphometry | | | | | | | |
| *n* (hearts) | 14 | 14 | 14 | 12 | 12 | 12 |  |
| HW/BW (mg/g) | 4.36 ± 0.18 | 4.20 ± 0.24 | 5.49 ± 0.57 ****^^**## | 4.459 ± 0.15 | 5.094 ± 0.29 ‡ | 4.503 ± 0.44 |  |
| LVM (mg) | 85.39 ± 0.23 | 84.17 ± 0.59 | 134.85 ± 0.81 ****^^**## | 88.85 ± 0.32 | 128.52 ± 0.67 ‡ | 86.33 ± 0.46 |  |
| Echocardiography | | | | | | | |
| *n* (hearts) | 10 | 10 | 10 | 11 | 11 | 10 |  |
| IVS; d (mm) | 0.731 ± 0.05 | 0.732 ± 0.08 | 0. 862 ± 0.20 ***^**# | 0.710 ± 0.08 | 0.823 ± 0.12 | 0.712 ± 0.18 |  |
| IVS; s(mm) | 1.101 ± 0.08 | 1.103 ± 0.11 | 1.325 ± 0.11 ****^**# | 1.119 ± 0.14 | 1.301 ± 0.09 | 1.120 ± 0.07 |  |
| LVID; d (mm) | 3.730 ± 0.14 | 3.761 ± 0.14 | 4.872 ± 0.44 ****^**# | 4.247 ± 0.51 | 4.431 ± 0.65 ‡ | 4.087 ± 0.43 |  |
| LVID; s (mm) | 2.395 ± 0.32 | 2.401 ± 0.12 | 3.900 ± 0.43 ****^**# | 2.978 ± 0.73 | 3.240 ± 0.32‡ | 2.812 ± 0.37 |  |
| LVPW; d (mm) | 0.742 ± 0.13 | 0.751 ± 0.14 | 1.038 ± 0.24****^^**## | 0.775 ± 0.26 | 0.848 ± 0.28 ‡ | 0.779 ± 0.16 |  |
| LVPW; s (mm) | 1.031 ± 0.17 | 1.028 ± 0.21 | 1.484 ± 0.32 ****^^**## | 0.944 ± 0.42 | 1.165 ± 0.18 ‡ | 0.981 ± 0.06 |  |
| Electrocardiography | | | | | | | |
| *n* (hearts) | 10 | 10 | 10 | 11 | 11 | 10 |  |
| QRS Interval (s) | 0.00988±0.00089 | 0.0105±0.00051 | 0.0131±0.00053** | 0.0127±0.00021**ǂǂ** | 0.0117±0.00078 | 0.0108±0.00014 |  |
| QT Interval (s) | 0.021 ± 0.002 | 0.019 ± 0.002 | 0.035 ± 0.013 **## | 0.034 ± 0.004 **ǂ** | 0.028 ± 0.015 ‡ | 0.024 ± 0.002 |  |
| QTc (s) | 0.056 ± 0.005 | 0.051 ± 0.005 | 0.081 ± 0.010 *# | 0.074 ± 0.012 **ǂ** | 0.080 ± 0.011 ‡ | 0.053 ± 0.006 |  |
| T Amplitude (mV) | 0.470 ± 0.043 | 0.427 ± 0.023 | 0.188 ± 0.074±*## | 0.263 ± 0.070**ǂ** | 0.256 ± 0.038 ‡ | 0.432 ± 0.029 |  |
| ST Height (mV) | 0.35 ± 0.072 | 0.33 ± 0.081 | 0.18 ± 0.054*# | 0.22 ± 0.042**ǂ** | 0.21 ± 0.064‡ | 0.31 ± 0.061 |  |
| JT Interval (s) | 0.007604±0.00058 | 0.007324±0.00018 | 0.02471±0.0020**## | 0.02206±0.0015**ǂǂ** | 0.01446±0.0023‡‡ | 0.009042±0.00049 |  |
| P Amplitude (mV) | 0.08800±0.013 | 0.08102±0.0074 | -0.1047±0.032**## | 0.03493±0.063 | 0.05726±0.0064 | 0.08002±0.0067 |  |
| PR Interval (s) | 0.03854±0.0011 | 0.03971±0.00063 | 0.04378±0.00070*# | 0.04188±0.00071 | 0.04140±0.0013 | 0.03885±0.0033 |  |

**Table S2:** **Quantitative data of morphometry, echocardiography and electrocardiography**

**Table S2:** **HW**, heart weight; **BW**, body weight; **LVM**, left ventricle mass; **IVSd**, Interventricular septal thickness diastolic; **IVSs**, Interventricular septal thickness systolic; **LVIDd**, left ventricular internal diameter diastolic; **LVIDs**, left ventricular internal diameter systolic; **LVPWd**, left ventricular posterior wall thickness diastolic; **LVPWs**, left ventricular posterior wall thickness systolic; **QTc**, Corrected QT Interval. Data are expressed as mean ± SD. P values < 0.05 were deemed significant. *p<0.05, **p<0.01 PCH vs Vhl/Ctrl; **^**p<0.05, **^^**p<0.01 PCH vs ISO+ALX; # p<0.05, ##p<0.01 PCH vs ISO+ALX+FSK; **ǂ**p<0.05, **ǂǂ**p<0.01 ISO+ALX vs Vhl/Ctrl; ‡p<0.05 ISO+FSK vs Vhl/Ctrl.
